# Implant-supported canine retraction using different reactivation intervals of elastomeric chains: A CBCT-based split-mouth randomized controlled trial

**DOI:** 10.1590/2177-6709.28.5.e2123166.oar

**Published:** 2023-11-03

**Authors:** Hend S. ELSAYED, Amr R. EL-BEIALY, Reem ALSHAZLY, Ahmad ALMOHAMMAD, Karim ELAZAB, Rodayna EL-BADAWY, Juan Martin PALOMO, Yehya A. MOSTAFA

**Affiliations:** 1 Oral and Dental Research Institute, National Research Centre, Department of Orthodontics & Pediatric Dentistry (Giza, Egypt).; 2 Cairo University, Faculty of Dentistry, Department of Orthodontics and Dentofacial Orthopedics (Cairo, Egypt).; 3 Future University in Egypt, Faculty of Oral & Dental Medicine, Department of Orthodontics and Dentofacial Orthopedics (Cairo, Egypt).; 4 Case Western Reserve University, School of Dental Medicine, Department of Orthodontics (Cleveland/OH, USA).

**Keywords:** Canine retraction, Appointment interval, Reactivation interval, Rate of tooth movement, Root resorption

## Abstract

**Introduction:**

Orthodontists attempt to maximize treatment efficiency regarding time, tooth position and adverse effects. A new approach, not yet explored, is the activation frequency.

**Objective:**

The aim of this split-mouth randomized controlled trial was to evaluate the effect of reactivation intervals on the efficiency of tooth movement.

**Methods:**

Thirty eight patients having a Class I malocclusion with bimaxillary dentoalveolar protrusion or severe crowding, Class II with mandibular deficiency or Class III, requiring first premolar extraction and canine retraction were recruited. Elastomeric chains producing 150g were replaced every two, four, six or eight weeks. There were 36, 37, 36, and 36 quadrants randomly allocated to these groups, respectively. The canine retraction rate was the primary outcome. Canine tipping, rotation, and root resorption and pain were the secondary outcomes. Only the outcome assessors were blinded to group assignment.

**Results:**

The average total movement for the 6 months was 5.14, 5.31, 2.79 and 3.85 mm for the two-week, four-week, six-week and eight-week reactivation intervals, respectively. Root resorption was significantly higher in the two-week and four-week groups. No adverse events were observed.

**Conclusion:**

The canine retraction rate, tipping, rotation and pain were similar in 2, 4, 6 and 8-week activation intervals groups. Longer reactivation intervals show less root resorption. The trial protocol was not pre-registered. The study was self-funded.

## RESUMO

**Introdução:**

Os ortodontistas buscam otimizar a eficiência do tratamento quanto ao tempo de duração, à posição dos dentes e aos efeitos adversos. Um aspecto ainda não avaliado são os diferentes intervalos entre as ativações.

**Objetivo:**

O objetivo desse estudo controlado e randomizado de boca dividida foi avaliar a influência de diferentes intervalos de reativação na eficiência da movimentação dentária.

**Métodos:**

Foram recrutados 38 pacientes com má oclusão de Classe I com biprotrusão dentoalveolar ou apinhamento severo, Classe II com deficiência mandibular ou Classe III, que necessitavam de extração do primeiro pré-molar e retração do canino. As cadeias elastoméricas gerando 150 g foram substituídas a cada duas, quatro, seis ou oito semanas, constando 36, 37, 36 e 36 quadrantes alocados aleatoriamente nesses grupos, respectivamente. O desfecho primário foi a taxa de retração do canino. Os desfechos secundários foram a inclinação, a rotação e a reabsorção radicular do canino, e a dor. Somente os avaliadores dos resultados não tinham conhecimento da alocação nos grupos.

**Resultados:**

O movimento total médio para os seis meses foi de 5,14; 5,31; 2,79 e 3,85 mm para os intervalos de reativação de duas semanas, quatro semanas, seis semanas e oito semanas, respectivamente. A reabsorção radicular foi significativamente maior nos grupos de duas e quatro semanas. Não foram observados eventos adversos.

**Conclusão:**

A taxa de retração, a inclinação e a rotação do canino e a dor foram semelhantes nos grupos com intervalos de ativação de duas, quatro, seis e oito semanas. Intervalos de reativação mais longos mostram menos reabsorção radicular. O protocolo do estudo não foi pré-registrado. O estudo foi autofinanciado.

## INTRODUCTION

Orthodontic treatment time can range between 18 and 45 months, with an average of 24 months.^1^ The treatment time may extend in extraction cases, depending on the number of extracted teeth.^2^ The space closure and adjacent teeth uprighting may take up to 10 months.^3^

Systematic reviews^4^ ^,^ ^5^ reported that different methods of canine retraction have similar rates. A factor not yet explored is the reactivation interval. Varying the duration between appointments may affect the onset and duration of the resorptive and depository cycles of bone remodeling.

Generally, appointment intervals are guided by the type of appliance and the patient’s and clinicians’ schedules. The JCO readers’ survey^6^ and the AAO survey^7^ report that the most frequent appointment intervals is about 6 weeks. Fewer orthodontists were seeing patients every 4 weeks.^6^ Nowadays, appliances sustain adequate forces and support less frequent visits.

A retrospective study by Alger^8^ showed that extending the appointment interval to six weeks did not prolong the overall treatment time.

Decreasing the number of appointments may improve patient satisfaction, decrease orthodontists’ chairside time, indirect cost and allow more patients to be scheduled.^5^ However, it may cause overcorrection and delay monitoring patient’s oral hygiene and cooperation. 

## AIM AND HYPOTHESIS

The research question was: in orthodontic patients undergoing canine retraction, would replacing the elastomeric chain every two, six, or eight weeks, compared to four weeks, provide a faster rate of retraction? The null hypothesis was that there would be no difference in the rate of canine retraction between the different reactivation intervals.

## MATERIAL AND METHODS

### TRIAL DESIGN AND SETTING

The trial was conducted between April 2017 and February 2019. The institutional review board’s approval (Identification no.: 20153110-(8)14-2017) and participants’ consent were obtained before the treatment.

In this split-mouth randomized controlled trial, quadrants were allocated to one of the four experimental groups. The allocation ratio was 1:1:1:1.

### PARTICIPANTS AND ELIGIBILITY CRITERIA

Fifty consecutive patients attending the orthodontic outpatient clinic were screened. Thirty-eight patients (30 females) were recruited, with an age range between 15.5 and 23.5 years. Four premolars were extracted in 33 patients, three premolars in 3 patients and two premolars in 2 patients. The trial included 145 quadrants (74 maxillary and 71 mandibular). At the start, there were 36, 37, 36, and 36 quadrants in the two-week, four-week, six-week and eight-week groups, respectively. The inclusion criteria were: permanent dentition, malocclusion requiring at least two first premolar extractions, and Group A anchorage demand. The recruited patients had Class I malocclusion with severe crowding or bimaxillary dentoalveolar protrusion, Class II div. 1, or Class III. Patients were excluded if pregnant, smokers, or reported a systemic disease or medication that interfered with bone metabolism. Patients with a history of dentofacial anomalies, periodontal disease, or orthodontic treatment were not eligible.

### INTERVENTIONS

Upper and lower first molars were banded (0.022-in bands: American Orthodontics, Sheboygan, Wis.). Canines and second premolars were bonded with 0.022-in Roth prescription brackets (Mini Master; American Orthodontics, Sheboygan, USA). Leveling and alignment were performed using sequential wires up to 0.016 x 0.022-in SS, bypassing the four incisors.

**Figure 1:**
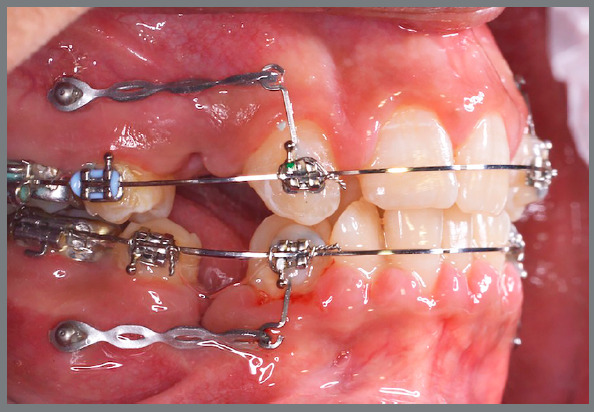
Canine retraction with elastomeric chains connected from the miniscrews to the power arms.

Temporary anchorage devices (TADs; 1.8mm x 8mm) (3M Unitek, Monrovia, CA) were placed between the second premolars and first molars. The patients were then referred for premolars’ extraction and canine retraction was started within one to two weeks. Power arms, 8mm in length, were fabricated using a 0.016 x 0.016-in stainless steel wire to approximate the delivered force to the center of resistance (Fig 1).

A pre-retraction cone beam computed tomography (CBCT; Aceton X-mind Trium. La Cietat. France) was acquired within two weeks of the premolar extraction. A second CBCT was acquired after 6 months of canine retraction. Patients who missed the follow-up CBCT were rescheduled within 7 days. The DICOM images were imported into InVivo 5 software (version 5.3.1; Santa Clara, CA, USA).

Using a force gauge (50-500 g, Dental Morelli Ltda, Sorocaba, São Paulo, Brazil), the elastomeric chains (Short, Silver, American Orthodontics, Sheboygan, Wis.) were stretched between the TADs and the power arms, to deliver 150g. According to the allocated intervention, the elastomeric chains were replaced every two, four, six or eight weeks. It was possible for a single patient to have a different reactivation schedule for each quadrant. The TADs stability was checked at each visit.

**Figure 2:**
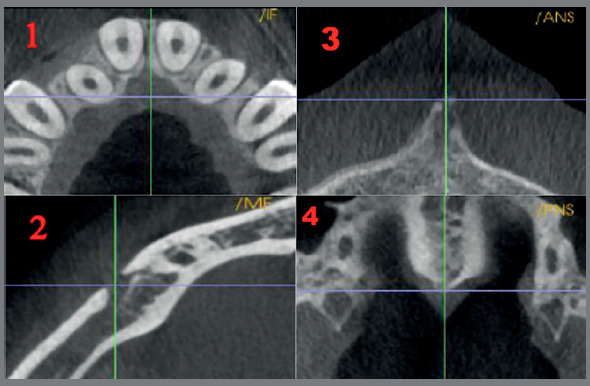
Bony landmarks: 1. Incisive foramen, 2. Mental foramen, 3. Anterior Nasal Spine, 4. Posterior Nasal Spine.

Using the Invivo 5 software, CBCT landmarks were identified on the 3D volume and adjusted on the multiplanar slice locator, in the axial, sagittal and coronal views, using the Invivo 5 software. The stable bony landmarks; incisive foramen, the anterior and posterior nasal spines and the right and left mental foramina were identified (Fig 2).

**Figure 3:**
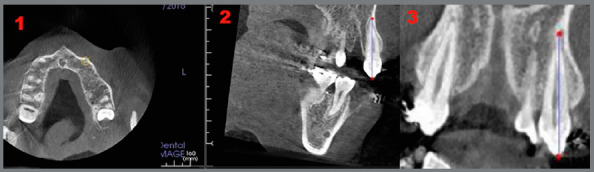
Localization of canine root apex in transverse (1), sagittal (2) and coronal (3) planes. CBCT reference planes (Medial Sagittal Plane, Horizontal Plane, maxillary & mandibular frontal planes ).

Dental landmarks, the canine crown tip and canine root apex were localized in the axial, sagittal and coronal planes. They were identified as the smallest point at the apex, which coincided in the three planes (Fig 3). Root resorption was calculated as the difference in canine length, from cusp tip to root apex, between the pre- and post-retraction CBCTs.

**Figure 4:**
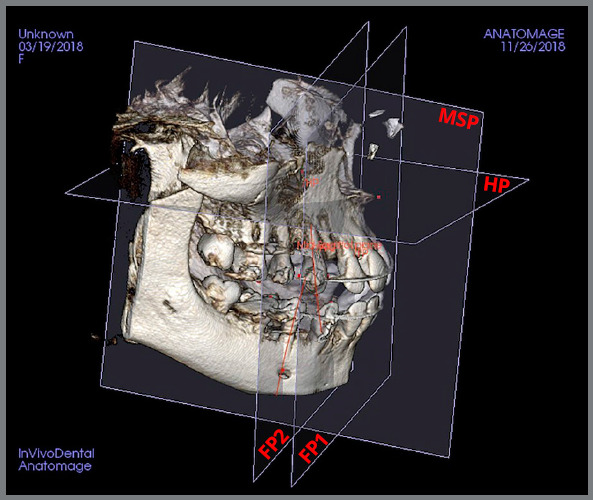
CBCT reference planes: Median Sagittal Plane (MSP), Horizontal Plane (HP), Maxillary (FP1) and Mandibular (FP2) Frontal Planes.

Four reference planes were constructed. The median sagittal plane (MSP) was constructed along the anterior nasal spine, the incisive foramen and the posterior nasal spine. The horizontal plane (HP) was constructed perpendicular to the MSP and passing through the Anterior Nasal Spine (ANS) and the Posterior Nasal Spine (PNS). The maxillary frontal plane (FP1) was perpendicular to the MSP and the HP, passing through the incisive foramen. Similarly, the mandibular frontal plane (FP2) was perpendicular to the MSP and the HP, passing through the right and left mental foramina. The planes are shown in Figure 4.

The primary outcome was the amount of canine retraction for six months. The amount of canine retraction was calculated as the difference between the pre-retraction and post-retraction perpendicular distances from the upper and lower canine cusp tips to the constructed FP1 and FP2, respectively. The amount of canine retraction for the six months was reported. Secondary outcomes of interest were canine tipping, rotation, root resorption and pain. Canine tipping was measured between the long axis of the canine (cusp tip to root apex) and their respective frontal planes (Fig 4). The maxillary and mandibular canine rotation angles were measured from a line connecting the maximum mesial and distal crown convexities to their respective frontal planes.

The outcome evaluator remeasured 10 CBCTs, to calculate the intra-observer agreement. A second investigator measured the same records, and inter-observer agreement was calculated.

Patients were asked to report their level of pain USING a 100-mm Visual Analogue Scale^9^ (VAS). Pain was scored for the first 10 days after every activation during the 6 months of retraction.

### SAMPLE SIZE CALCULATION

A pilot study with 10 patients was conducted. The means and standard deviations of canine retraction (in mm) for the two, four, six and eight-week reactivation were 0.51±0.43, 0.64±0.25, 0.58±0.41, 0.90±0.41, respectively. The sample size using the means for six months of retraction was calculated using the repeated measure ANOVA in the G*Power v. 3.1.9.6 software, with a 95% CI and 0.8 power. A total sample size of 36 patients was estimated.

### RANDOMIZATION AND ALLOCATION CONCEALMENT

Each of the 145 quadrants was randomized evenly to the 2, 4, 6, or 8-week reactivation intervals, using the combined CHOOSE and RAND functions in Microsoft Office Excel Mac (v. 16.24; Microsoft, Redmond, USA). The additional quadrant was in the four-week group. The computer-generated allocation sequence was held by the principal investigator, and the clinician was informed of the allocation at the time of retraction.

### BLINDING

It was not possible to mask the patients and operators. The outcome assessors were blinded and the measurements were performed for unidentified CBCTs.

### STATISTICAL ANALYSIS

The data were evaluated for normality using the Shapiro-Wilk and Kolmogorov-Smirnov tests. The intraclass correlation coefficients (ICC) were calculated for the inter-observer agreement. The descriptive data were presented as medians and interquartile ranges.

The dependent nature of the groups was analyzed using Friedman’s and Wilcoxon’s tests. The Friedman test compared the canine retraction rate, tipping, rotation, and root resorption between the groups. Pairwise comparisons of the two, six, eight-week groups and the control group were performed using Wilcoxon’s test. The Cochran Q test compared the percent of patients reporting pain in the groups. The significance level was set at *p*≤0.05 for two-tail tests. Statistical analysis was performed with IBM SPSS Statistics for Windows (Version 23.0. Armonk, NY: IBM Corp.).

## RESULTS

**Figure 5:**
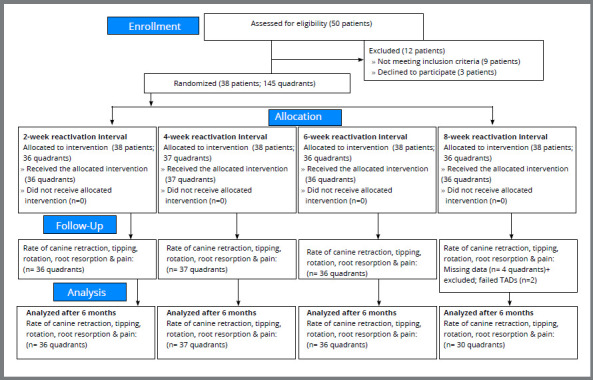
CONSORT flow chart of patient participation.

Thirty-eight patients completed the trial. After six months of canine retraction, the data of 36, 37, 36, and 30 quadrants were analyzed. Data for 6 quadrants were excluded from the eight-week group. This was due to the failure of two mandibular miniscrews and missing data for four quadrants. The CONSORT flow chart (Fig 5) shows the patients’ progress throughout the trial.

All outcomes showed a non-normal distribution. The median and interquartile range for each group are reported in Table 1.

**Table 1:** Descriptive statistics and comparison between the groups for canine retraction after 6 months.

	Two weeks (36)	Four weeks (37)	Six weeks (36)	Eight weeks (30)	p-value	Effect size
	Median (Q1 - Q3)	Median (Q1 - Q3)	Median (Q1 - Q3)	Median (Q1 - Q3)		
^†^ Canine retraction rate (mm/6 month)	5.14 (1.64-6.35)	5.31 (2.55-6.72)	2.79 (1.77-5.19)	3.85 (2.05-3.85)	0.615	0.02
^†^ Canine tipping (degrees)	-6.82 (-11.12- -3.13)	-8.15 (-10.8- -3.6)	-4.93 (-7.95- -0.3)	-5.66 (-10.33- -0.54)	0.066	0.08
^†^ Canine rotation (degrees)	11.26 (4.27-18.6)	10.71 (6.32-21)	8.68 (3.63-17.36)	9.27 (1.42-21.12)	0.632	0.019
^†^ Canine root resorption (mm)	1.13 (0.63-1.47)	1.09 (0.66-1.46)	0.57 (0.38-0.92)	0.62 (0.3-0.99)	0.002*	0.164

^†^ Friedman’s test, * Statistically significant at *p* ≤ 0.05.

The difference between the groups was insignificant for retraction rate, canine tipping and canine rotation after 6 months (Table 1).

There was a significant difference between the groups in terms of root resorption (Table 1). Pairwise comparisons showed significantly higher root resorption in the two and four-week groups, compared to the others (Table 2). 

**Table 2:** Descriptive statistics and comparison of changes between the two, six and eight-week reactivation intervals, and the control group (four-week).

	Comparison		Difference	95% CI	
	Control	Intervention		upper	lower
^†^ Canine retraction rate (mm/ 6 months)	4-week interval	2-week	-0.04	-1.18	1.25
		6-week	-0.99	-2.45	0.62
		8-week	-0.7	-1.63	0.54
^†^ Canine tipping (degrees)	4-week interval	2-week	1.25	-2.11	4.88
		6-week	2.55	-0.37	5
		8-week	1.22	-2.18	4.84
^†^ Canine rotation (degrees)	4-week interval	2-week	-1.94	-9.26	3.63
		6-week	-3.02	-9.25	2.73
		8-week	-5.26	-10.93	1.83
^†^ Canine root resorption (mm)	4-week interval	2-week	0.01	-0.29	0.25
		6-week	-0.39	-0.65	-0.13
		8-week	-0.4	-0.71	-0.03

^†^ Wilcoxon test for pairwise comparisons.

Most patients reported zero pain for the 10 days following canine retraction (Table 3). No statistical difference was shown between the groups for the percent of patients reporting pain (Table 4). 

**Table 3:** Descriptive statistics of collective pain scores for the 10 days following activations.

Time	Two weeks	Four weeks	Six weeks	Eight weeks
	Median (Q1- Q3)	Median (Q1- Q3)	Median (Q1- Q3)	Median (Q1- Q3)
Day 1	27 (5.5-32.5)	28.8 (22.6-44)	27.5 (6-30)	27.5 (22.3-68.8)
Day 2	12 (3-37)	26.5 (16-42.5)	24.5 (2)^†^	26.5 (13.5-56.5)
Day 3	24.8 (7)^†^	1.5 (1)^†^	3.5 (2)^†^	22.8 (4)^†^
Day 4	21.3 (2.5)^†^	40.5^††^	40.5^††^	40.5^††^
Day 5	34^††^	0	0	0
Day 6	32.8 (30.5)^†^	0	0	20^††^
Day 7	0	0	0	20^††^
Day 8	18^††^	0	19.5^††^	0
Day 9	0	0	0	0
Day 10	0	0	0	0

^†^ Q3 was not computed for two cases with pain.

^††^ IQR was not computed for one case with pain.

**Table 4:** Descriptive statistics and comparison between the percent of patients reporting pain in the four groups.

Time	Two weeks (n = 36)	Four weeks (n = 37)	Six weeks (n = 36)	Eight weeks (n = 30)	p-value
	%	%	%	%	
Day 1	14	27	14	16.7	0.815
Day 2	14	29.7	5.6	16.7	0.096
Day 3	5.6	8.1	8.3	6.6	0.682
Day 4	5.6	2.7	2.8	3.3	0.801
Day 5	2.8	0	0	0	Not computed^§^
Day 6	5.6	0	0	3.3	0.392
Day 7	0	0	0	3.3	0.392
Day 8	2.8	0	2.8	0	0.572
Day 9	0	0	0	0	Not computed^§^
Day 10	0	0	0	0	Not computed^§^

^§^ not computed in the absence of patients with pain in all quadrants for that day.

^*^ Statistically significant at *p* ≤ 0.05.

The inter-observer agreement (ICC) and confidence intervals (CI) were (0.989; 0.969-0.996), (0.939; 0.821-0.98), (0.870; 0.66-0.954) and (0.999; 0.9995-0.9999) for canine retraction, tipping, rotation and root resorption, respectively.

## DISCUSSION

Studies have previously compared different methods of canine retraction. Most studies concluded that different techniques produce similar rates of movement.^4^ Thus, the commonly used elastomeric chains are as effective, and perhaps more economic than other means.^5^ ^,^ ^10^

However, force decay of elastomeric chains is a disadvantage. Studies showed a significant decrease in the initial force during the first hour of activation. It was then relatively stable for the following four weeks, retaining 30-60% of the force.^11^ This evidence led clinicians to replace the elastomeric chains every 3-4 weeks.^12^

Studies reported canine retraction for up to 15^13^ or 16^14^ weeks with unchanged elastomeric chains. Therefore, the elastomeric chains were chosen for this study.

The 2005 JCO survey^6^ evaluated clinicians^’^ preference regarding interval between appointments. None of the responders indicated appointment intervals of three weeks or less. A period of six to eight weeks between appointments was preferred in non-extraction cases,^15^ extraction cases using friction mechanics and in working parents and school children.^6^ Yet, the literature search performed in the present study showed a four-week reactivation interval in most of the studies using elastomeric chains.

Varying the reactivation interval for canine retraction produced similar amounts of tooth movement. The difference between the highest and lowest amount was 2.35mm, which was statistically insignificant. This may be explained by the variation in the amount of canine retraction and the fact that tooth movement depends on the strain and tissue reaction produced in the periodontium, regardless of the reactivation rate.

Although orthodontic appliances were activated every two weeks^8^ ^,^ ^15^ in the past, there are no trials evaluating the effect of this interval. However, studies by Ziegler and Ingervall^16^ and Al-Suleiman and Shehadah^17^ used a three-week reactivation interval, and showed average monthly canine retraction rates of 1.41mm and 1.42mm, respectively. Lotzof et al.^18^ reported 1.63 mm of retraction every three weeks. The higher retraction rates observed with frequent activations may be explained by the use of 0.018-in stainless steel wires, which may have reduced friction during sliding.

Studies that reactivated elastomeric chains every four weeks observed canine retraction comparable to the results of the present study. Mezomo et al.^19^ showed 2.53±0.62 mm of canine retraction in three months (0.84±0.21 mm/month), while Dixon et al.^10^ reported 2.33±1.18 mm of overall space closure in four months (0.58mm/month).

Mitra et al^20^ reactivated power chains every six weeks, and reported a total canine retraction of 2.78±0.13 mm in 4.5 months (0.61mm/month). This rate was closer to that shown by Dixon et al.^10^ Compared to the results of the present study, the slower rate of space closure in these trials may be explained by the use of posted archwires instead of direct attachment to the canine brackets.

Heavy archwires are recommended for sliding mechanics, to prevent wire bending and binding with the brackets.^21^ However, static friction is largely influenced by the vertical dimension of the archwire.^22^ Approximately half of the retraction force is lost due to this friction.^23^ Therefore, we retracted the canines using 0.016 x 0.022-in archwires. This wire’s flexibility may increase the kinetic friction by allowing a larger angle between the wire and the bracket. To overcome this, we used a relatively low force for retraction,^23^ and a power arm^21^ ^,^ ^24^ was used to approximate the line of action of the force to the center of resistance of the tooth,^25^ thus minimizing wire bending. Several researchers have used 0.016 x 0.022-in archwires^24^ ^,^ ^26^ and even 0.018-in archwires,^16^ to reduce the friction during sliding. 

Bypassing the four incisors avoided round-tripping the anterior teeth, and prevented these teeth to take up the extraction space in the cases with crowding. It also allowed the early retraction of the canines.

It was expected that frequent activations would increase the canine tipping. However, the four groups showed similar tipping, probably due to the use of the power arms. Studies by Zeigler and Ingervall,^16^ and Al-Suleiman and Shehada^17^ lasted six and five months, respectively. The tipping of 8.5° (1.41±1.29°/month) reported by Zeigler and Ingervall^16^ was similar to that observed in the four-week reactivation interval of the present study; while Al-Suleiman & Shebada^17^ reported 14° of tipping. This result was probably due to the less rigid archwire used with the 0.018-in slot brackets.

Zeigler and Ingervall^16^ and Mezomo et al.^19^ reported 4.04° and 4.8° of canine rotation for every millimeter of retraction, respectively. Total rotation was 24° in six months and 12.27° in three months. Al-Suleiman and Shehadah^17^ reported 3.32±1.42° of rotation. However, it is not clear if this rotation was for the five months of retraction or per millimeter of retraction. In this study, an average of 2° of canine rotation per millimeter of retraction was comparable to the other studies. However, canine rotation showed high variation in this sample.

The studies employed different methods of outcome measurements. Three studies used innovative tools to measure the amount of canine retraction, without radiation exposure^16^ ^,^ ^18^ ^,^ ^19^ The pre-retraction model was used to construct a plate, with occlusal indentations for the molars and incisors.^16^ This plate was fitted intra-orally during retraction. The distal surface of the canine bracket was measured to a stiff vertical wire incorporated in the plate. The authors reported that the molars moved during the canine retraction and that the plate had to be modified to fit at the incisal edges. Other studies constructed an acrylic transfer plate, on the rugae area, with stiff wire extensions.^18^ ^,^ ^19^ The plate was transferred to subsequent models, and the canines^21^ and the first molars^18^ ^,^ ^19^ were measured to the wire extensions. Although the studies used different methodology, their results did not show large variations, and the reporting of the mean difference makes comparing the results possible. The use of unstable teeth for reference may be adequate when assessing overall space closure.^10^ ^,^ ^20^ However, it will not provide information on the amount of space closed by anchorage loss of the posterior unit.

Orthodontic treatment produces mild to moderate root shortening. Frequent reactivations decrease the time for the resorbed cementum to recover. The maximum root resorption in all the groups was smaller than 2.7 mm, which is within the average amount reported for orthodontic apical root resorption. The results in this study were higher than those reported by Alkebsi et al^27^ (0.73mm). Although they used similar mechanics for retraction, their trial duration lasted three months. Longer durations of tooth movement were associated with increased root resorption.^28^ The progression of root resorption should be monitored if the force is frequently reactivated.

In the present study, the follow-up CBCTs were acquired after six months of retraction. This left a waiting period between the complete canine retraction and the acquisition of the second CBCT for the 27 canines, which may have allowed for the correction of root resorption, distal tipping and rotation, thus underestimating the true amounts of change. However, this does not seem to be the case in the present study, since the changes observed were similar to those reported in the above-mentioned studies.

Similar to other studies,^9^ ^,^ ^29^ the pain intensity was highest on the first day after activation, then declined. The pain scores showed high inter and intra-individual variability, which was also reported by other studies evaluating canine retraction.^30^ It was not possible to statistically compare the pain intensity between the groups, due to the high frequency of patients reporting zero pain. The percentage of patients who reported pain in the four groups was similar. Yet, compared to the other groups, pain lasted for more days in the two-week reactivation group. Pain was rarely reported in patients participating in this study, which differs from other studies.^9^ ^,^ ^29^ ^,^ ^30^ This may indicate that ethinicity and environment play a strong role in the pain experience.^31^ ^,^ ^32^

The results of the present study should be interpreted with caution. Limitations of the study, including the missing data reported in the eight-week group and the uneven number of quadrants across the groups, may affect the robustness of the analysis. Block randomization with each patient receiving the four interventions would have eliminated this problem. However, this was not possible since not all patients required four premolar extraction. The large variability for canine tipping, rotation, root resorption, and pain should be considered. Some image artifacts in the CBCTs prevented a clear localization of the mesial and distal crown convexities, which may have increased the error of measurements for canine rotation. Although the trial protocol was not pre-registered, the authors adhered to the protocol and have reported all relevant outcomes.

Nineteen quadrants in seven patients had severe crowding. Although the anterior teeth were not leveled and aligned, they may have used up part of the extraction space. The inclusion of these cases may have underestimated the amount of canine retraction. 

There are some drawbacks regarding the generalizability of this study. The pain experience reported is different from most published trials, which may be attributed to ethnicity and culture. There was an unequal representation of males and females in the present study. Since this was the first prospective trial to evaluate the effect of reactivation intervals, further studies are required to elucidate the reliability of these results.

This study was conducted to highlight the effect of different reactivation intervals on the canine retraction rate using conventional elastomeric chains. The present data may guide the methodology and sample size calculation of future studies.

## CONCLUSIONS

There was no significant difference in the canine retraction rate, tipping, rotation and pain, when comparing two, four, six, and eight-week activation intervals. Longer reactivation intervals showed less root resorption.
